# Unraveling the Complexity of Imported Malaria Infections by Amplicon Deep Sequencing

**DOI:** 10.3389/fcimb.2021.725859

**Published:** 2021-09-14

**Authors:** Xi He, Daibin Zhong, Chunyan Zou, Liang Pi, Luyi Zhao, Yucheng Qin, Maohua Pan, Siqi Wang, Weiling Zeng, Zheng Xiang, Xi Chen, Yanrui Wu, Yu Si, Liwang Cui, Yaming Huang, Guiyun Yan, Zhaoqing Yang

**Affiliations:** ^1^Department of Pathogen Biology and Immunology, Kunming Medical University, Kunming, China; ^2^Program in Public Health, College of Health Sciences, University of California at Irvine, Irvine, CA, United States; ^3^Department of Electrocardiogram, Guangxi Zhuang Autonomous Region People’s Hospital, Nanning, China; ^4^Department of Infectious Diseases, Shanglin County People’s Hospital, Shanglin, China; ^5^Department of Cell Biology & Genetics, Kunming Medical University, Kunming, China; ^6^Department of Internal Medicine, Morsani College of Medicine, University of South Florida, Tampa, FL, United States; ^7^Department of Protozoa, Guangxi Zhuang Autonomous Region Center for Disease Prevention and Control, Nanning, China

**Keywords:** imported malaria, mixed-species infection, relapse, recrudescence, multiplexity of infection, drug resistance

## Abstract

Imported malaria and recurrent infections are becoming an emerging issue in many malaria non-endemic countries. This study aimed to determine the molecular patterns of the imported malaria infections and recurrence. Blood samples were collected from patients with imported malaria infections during 2016–2018 in Guangxi Zhuang Autonomous Region, China. Next-generation amplicon deep-sequencing approaches were used to assess parasite genetic diversity, multiplexity of infection, relapse, recrudescence, and antimalarial drug resistance. A total of 44 imported malaria cases were examined during the study, of which 35 (79.5%) had recurrent malaria infections within 1 year. The majority (91.4%) had one recurrent malaria episode, whereas two patients had two recurrences and one patient had three recurrences. A total of 19 recurrence patterns (the species responsible for primary and successive clinical episodes) were found in patients returning from malaria epidemic countries. Four parasite species were detected with a higher than usual proportion (46.2%) of non-falciparum infections or mixed-species infections. An increasing trend of recurrence infections and reduced drug treatment efficacy were observed among the cases of imported malaria. The high recurrence rate and complex patterns of imported malaria from Africa to non-endemic countries have the potential to initiate local transmission, thereby undermining efforts to eliminate locally acquired malaria. Our findings highlight the power of amplicon deep-sequencing applications in molecular epidemiological studies of the imported malaria recurrences.

## Introduction

Malaria remains a major public health problem in sub-Saharan Africa. In 2019, an estimated 229 million cases and 409,000 deaths of malaria occurred globally with the vast majority of cases (94%) in the African region, followed by South-East Asia (3%) (WHO, 2020). In addition, imported malaria infection is emerging in many malaria-free countries including China, which have recently become malaria-free. Historically, the Guangxi Zhuang Autonomous Region was one of the four highest malaria-endemic areas in southern China (Yang et al., 2018). Guangxi had no indigenous malaria cases reported since 2013. Imported malaria is increasing due to increasing travel for tourism and trade in high-risk areas; Guangxi remains the area of China at highest risk for imported malaria (Lin et al., 2017; Jianhai et al., 2020). During 2011 and 2018 in Guangxi, a total of 3,943 imported malaria cases were reported and most of them originated from Africa (e.g., Ghana, Congo, and Cameroon), where *Plasmodium falciparum* is the predominant parasite species (Lin Kang-Ming et al., 2019). The increase in imported malaria cases poses a major challenge for the malaria elimination program in China (Lai et al., 2019).

Accurate identification and assessment of imported malaria parasite multiplicity of infection (MOI), genetic diversity in relapse and recrudescence, and antimalarial drug resistance are essential for malaria treatment, prognosis, and prevention of malaria transmission. To improve control, we require information about the parasite’s diversity, transmission dynamics, relapse frequency, and mechanisms of adaptation to environmental and interventional pressures (Apinjoh et al., 2019). Traditional polymerase chain reaction (PCR)-based methods relying on DNA sequence length polymorphisms, such as antigenic polymorphic markers merozoite surface proteins (*msp1* and *msp2*), apical membrane antigen (*ama1*), circumsporozoite protein (*csp*), and microsatellites, have poor sensitivity for detection of less abundant parasite infections (Imwong et al., 2005; Kadekoppala and Holder, 2010; Lin et al., 2012; Lin et al., 2015), while next-generation sequencing of the PCR amplicon using Illumina HiSeq platforms provides a more sensitive molecular approach to determining the genetic diversity of *Plasmodium* infections and allows for more accurate identification and assessment. Amplicon deep sequencing (ADS) is more sensitive and reliable for detection of minority clone infections and determination of MOI (Flaherty et al., 2012; Early et al., 2019; Gruenberg et al., 2019), recrudescence and relapse (Lin et al., 2015; Zhong et al., 2018; Gruenberg et al., 2019), quantification of drug-resistance alleles (Bhatnagar et al., 2019; Brazeau et al., 2019; Gaye et al., 2020), and investigations of parasite microgeographic epidemiology (Koepfli and Mueller, 2017; Hemming-Schroeder et al., 2020).

This study was designed to answer the following questions: 1) What is the species composition of malaria parasites that cause imported malaria cases? 2) What proportion of these infections represent recrudescence or relapse? It is needed to establish whether the recurrent/relapsing infections were due to a new or existing infection. 3) Which mutations were associated with recrudescent infection in imported and recurrent episodes? To answer the first question, we conducted PCR and ADS using the mitochondrial mtDNA cytochrome b (CYTB) gene and small-subunit ribosomal (18S rRNA), referred to here as SSU gene markers for species identifications. To answer the second question, we performed ADS using *msp1* and *ama1* markers for understanding the genetic diversity between new and relapsing infections. Finally, we examined the gene mutations of the *P. falciparum* Kelch-domain protein (*pfk13*), chloroquine resistance transporter (*pfcrt*), and multidrug resistance protein-1 (*pfmdr1*) by Sanger sequencing. Since the effectiveness of primaquine (PQ) for the radical cure of vivax malaria is influenced by CYP2D6 activity (He et al., 2019), we wanted to determine whether the failure of PQ might be linked to CYP2D6 genotypes associated with poor metabolism of PQ. The cytochrome P450 isoenzyme 2D6 gene in *P. vivax* infected patients was also detected. 

## Materials and Methods

### Study Design

During 2016 and 2018, imported malaria infections in Shanglin County, Guangxi, were diagnosed by microscopic examination of Giemsa-stained thick and thin blood films. The questionnaires including gender, age, occupation, travel history, and date of diagnosis were recorded. Malaria patients were admitted for inpatient treatment following the guidelines of the Chinese Center for Disease Control and Prevention (CDC) and WHO recommendations on the diagnosis and treatment of uncomplicated and severe malaria (WHO, 2015) (**Supplementary Methods**). All patients who had two or more times malaria attack episodes were included in the study (**Supplementary Table 1**). The human subject protocol for this study was approved by the Shanglin Hospital Institutional Review Board. All participants supplied written informed consent.

### Sample Collection and Nested-PCR Amplification

Two hundred microliters of venous blood was collected from patients at the time of diagnosis at initial and repeated attacks. Malaria parasite DNA was extracted from whole blood using the High Pure PCR Template Preparation Kit (Roche, Mannheim, Germany) following the manufacturer’s instruction and eluted in 100 μl of water. The nested PCR method was used to identify the *Plasmodium* species with species-specific primers for *P. falciparum*, *P. vivax*, *P. malariae*, *P. ovale*, and *P. knowlesi* (Miguel-Oteo et al., 2017). The amplification reactions were conducted in 25 μl, containing 3 µl DNA template, 0.5 μM of each forward and reverse primer, 2 μl dNTP, 5 μl buffer, and 0.5 μl PrimeSTAR GAX DNA polymerase (Takara Bio Inc., Japan). The PCR amplification was performed at 94°C for 3 min, 35 cycles of 94°C for 30 s, 60°C for 30 s, and 72°C for 1 min, and a final extension at 72°C for 6 min. The amplified products were visualized using agarose gel electrophoresis.

### PCR Amplification and Deep Sequencing

Four target genes, including mitochondrial cytochrome b (CYTB) gene, small subunit ribosomal RNA (SSU) gene, the merozoite surface protein 1 (*msp1*) gene, and apical membrane antigen 1 (*ama1*) gene, were used to design seven sets of PCR primers based on the highly variable regions of SSU, CYTB, *pvmsp1*, *pvama1*, *pomsp1*, *pfmsp1*, and *pfama1* genes (**Supplementary Table 2**). PCR products (<400 bp) were prepared for next-generation sequencing by a two-step PCR approach following the previous reports (Lalremruata et al., 2017; Zhong et al., 2018). Briefly, the first-round PCR primers appended the overhang adapter sequence to the 5′ end of forward and reverse target gene-specific primers, while the second-round PCR used a universal primer with barcode primers that appended the 5′ end. Each sample was amplified in duplicate using a unique barcode (MIDs). The PCR primers and length of amplicon deep sequencing are listed in **Supplementary Table 2**. PCR amplification was performed in a total reaction volume of 25 μl reaction in duplicate following the protocols previously described (Zhong et al., 2018). *Plasmodium falciparum* laboratory strain 3D7 (MRA-102G) was used as control. Amplicons were cleaned and normalized to 1 ng/µl concentration using the SequalPrep Normalization Plate Kit (Thermo Fisher Scientific, Inc., Waltham, MA, USA). Amplicon deep sequencing was performed on an Illumina MiSeq platform in paired-end mode using a MiSeq Reagent Kit v3 PE300 (UCI Genomics High-Throughput Facility, Irvine, CA) with PhiX control (Illumina, PhiX Control v3) and the minimum target read depths of 10,000×.

### CYP2D6 Genotyping and Sequencing of Antimalarial Drug-Resistance Genes

The CYP2D6 gene in *P. vivax*-infected patients was analyzed by PCR amplification of the full-length CYP2D6-coding region using previously described primers and sequenced the PCR products (He et al., 2019). Patients’ CYP2D6 allele variants and the overall genotype activity score were evaluated by the CYP2D6 allele-naming database (www.Pharmvar.org/gene/CYP2D6). The full-length *pfk13* gene was amplified and sequenced using a previously published protocol (Zhang et al., 2016). The *P. falciparum* chloroquine resistance transporter gene (*pfcrt*) and the *P. falciparum* multidrug resistance protein-1 (*pfmdr1*) gene were amplified and sequenced using previously described primers (Li et al., 2015).

### Data Analysis

Deep-sequence data extraction, processing, and analyses were performed using the SeekDeep-targeted amplicon bioinformatics pipeline (Hathaway et al., 2017). Sequencing reads were separated by sample-specific barcodes and clustered according to markers, samples, and replicates. A cluster cutoff threshold of >2.0% in frequency, and a minimum read count of 10 for all markers were used to estimate haplotypes and frequency based on the accuracy of classification in positive controls and sample replicates. Classification of recurrences was conducted based on WHO classification of responses to treatment (WHO, 2009) and shared haplotypes between the initial and recurrent infections (Kyabayinze et al., 2003; Nyachieo et al., 2005). Analysis of haplotype and nucleotide diversity was performed using DnaSP v5. MEGA v7 was used to create a UPGMA phylogenetic tree. BioEdit v7 was used to align DNA sequences. All the case information was collected and input into SAS JMP 14.0 software (SAS Inc., Cary, NC), and statistical analyses were performed. The differences were compared by chi-square or Fisher’s exact tests at a two-sided p value <0.05 for statistical significance.

## Results

### Molecular Determination of the Malaria Parasite Species

**Figure 1:**
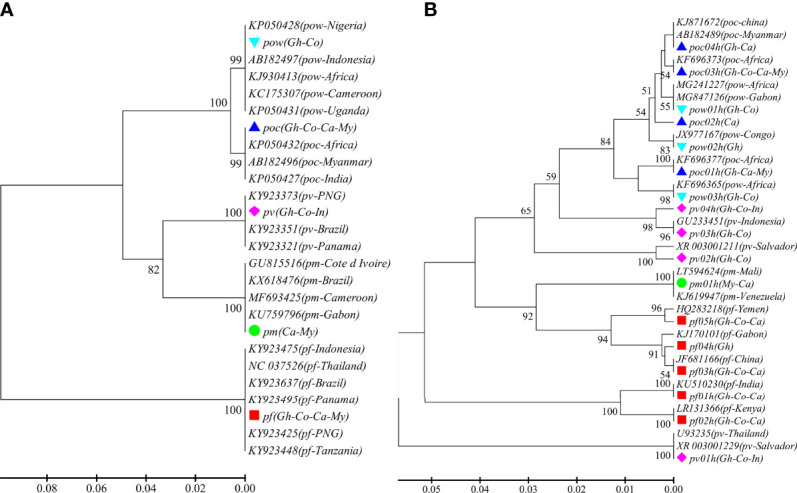
The phylogenetic UPGMA tree of haplotypes determined by amplicon deep sequencing. **(A)** CYTB phylogenetic tree for the 5 species and 21 sequences retrieved from GenBank (KY923637, KY923475, KY923495, KY923425, KY923448, NC_037526, KX618476, MF693425, GU815516, KU759796, KP050432, KP050427, AB182496, FJ409565, KJ930413, AB182497, KP050428, KP050431, KY923351, KY923321, KY923373). **(B)** SSU phylogenetic tree for the haplotypes of the 5 species and 19 sequences retrieved from GenBank (GU233451, JF681166, KJ170101, KJ619947, KU510230, MG241227, MG847126, U93235, XR_003001229, XR_003001211, LR131366, LT594624, KJ871672, AB182489, KF696373, KF696377, JX977167, KF696365, HQ283218). *pf*, *Plasmodium falciparum*; *pv*, *P. vivax*; *pm*, *P. malariae*; *poc*, *P. ovale curtisi*; and *pow*, *P. ovale wallikeri.* Different color markers represent different species identified in this study. *Gh*, Ghana; *Co*, Congo; *Ca*, Cameroon; *My*, Myanmar; and *In*, Indonesia.

A total of 93 samples from 44 patients were confirmed for malaria infections by nested PCR. All the patients were male between the ages of 22 and 60 years and had travel history from abroad with most of the destination countries (96.8%) in Africa. ADS of the CYTB and SSU markers was performed in 81 and 85 samples, respectively. An average sequencing coverage depth for the major haplotypes was 20,069 and 11,109 for CYTB and SSU markers, respectively (**Supplementary Table 3**). For CYTB, no polymorphism was detected within any species except for *P. ovale*, which includes two subspecies *P. ovale curtisi* and *P. ovale wallikeri*, whereas high polymorphism was found in the SSU marker with the number of haplotypes ranging from 1 to 5 in the five parasite species and similarity >99% compared to sequences in GenBank. The phylogenetic tree of CYTB comprises five clusters for the five *Plasmodium* species (**Figure 1A**), whereas a mixed pattern of SSU tree was observed in the five species (**Figure 1B**). Among the 93 samples, ADS identified 50 (53.8%) *P. falciparum*, 14 (15.1%) *P. vivax*, 9 (9.7%) *P. ovale curtisi*, 4 (4.3%) *P. ovale wallikeri*, 1 (1.1%) *P. malariae*, and 15 (16.1%) mixed-species infections (**Supplementary Table 4**). The proportion of *P. ovale curtisi* to *P. ovale wallikeri* is 2.25 (9/4) in single-species infections and 4.5 (9/2) in mixed-species infections. Six out of the 15 mixed species were identified by the SSU marker, which showed various frequencies of different haplotypes, and most of them had predominant *P. ovale curtisi* mixed with less abundant *P. falciparum* (**Supplementary Figure 1A, B**). The other mixed-species infections were determined by combined markers. The haplotype sequences of CYTB and SSU markers were deposited at NCBI GenBank with accession numbers MT942975–MT942996.

### Recurrent Species Patterns of Imported Malaria Cases in China

Among the 44 patients investigated, three patients (6.8%) had a second malaria attack within the first week of returning to China. Thirty-five patients (79.5%) had recurrent malaria infections later, but within 1 year of their return. The majority (91.4%) had one recurrent malaria episode, whereas two patients had two recurrences and one patient had three recurrences (**Table 1**). A total of 19 recurrence patterns were found in patients returning from malaria epidemic countries, including Ghana, Congo, and Cameroon. The average interval of malaria recurrence was 68.6 ± 11.6, ranging from 9 to 250 days. Overall, there were 26 (59.0%) not completely cured in all malaria species, 16 (36.4%) relapse in non-falciparum malaria, and 3 (6.8%) due to *P. falciparum* parasite dormancy or present at low-level parasitemia (Muehlenbachs et al., 2007; Malvy et al., 2018) (**Supplementary Table 5**). Among these, 27.2% (12/44) were recurrent infections. In addition, there were three patients who had tested positive malaria from 3 to 6 days, including two patients who showed to be coinfected with other species and six patients who had traveled abroad and repeated infections after previous malaria attack (**Supplementary Table 6**).

**Table 1:** Recurrence patterns of the imported malaria cases.

Pattern	*n*	Patient no.	Episode and interval of recurrence (days)				Remark	Country of Origin
			First	Second	Third	Fourth		
1	11	Pat01, 04, 07, 12, 15, 16, 17, 18, 21, 24, 33	*pf* ^#1^	*pf* (12–39)			11 *pf* ^F^	Ghana, Congo, Cameroon
2	2	Pat11, Pat25	*pf* ^#1^	*pf* (58–97)			2 *pf* ^D^	Ghana, Congo
3	1	Pat14	*pf* ^#1^	*pm* (36)			1 *pm* ^F^	Cameroon, Congo
4	1	Pat42	*pf* ^#1^	*pow*^#3^ (236)	*pow* (24)		1 *pow* ^R^*, 1 *pow* ^F^	Ghana
5	3	Pat03, Pat20, Pat27	*pf* ^#1^	*pf*+*poc* (15–17)			3 *pf* ^F^, 3 *poc* ^F^*	Ghana, Cameroon
6	3	Pat08, Pat19, Pat34	*pf* ^#1^	*poc* (71–103)			3 *poc* ^R^*	Ghana, Cameroon
7	1	Pat44	*pf* ^#1^	*pow* (74)			1 *pow* ^R^*	Congo
8	1	Pat35	*pf* ^#1^	*pv* (125)			1 *pv* ^R^*	Ghana
9	1	Pat30	*pf* ^#1^	*pv*+*poc* (31)			1 *pv* ^F^*, 1 *poc* ^F^*	Ghana
10	1	Pat36	*pv* ^#3^	*pv*^#3^ (66)	*pf*+*pv*^#3^ (108)	*pv* (313)	3 *pv* ^R^, 1 *pf* ^D^	Ghana
11	1	Pat31	*pv* ^#3^	*pow* (250)			1 pow ^R^	Ghana
12	2	Pat29, Pat39	*pv* ^#3^	*pv*^R^ (47–81)			2 *pv* ^R^	Cameroon, Congo
13	1	Pat22	*poc* ^#3^	*pf* (18)			1 *pf* ^F^	Ghana
14	1	Pat26	*pf+poc* ^#3^	*pf*+*poc* (21)			1 *pf* ^F^, 1 *poc* ^F^	Ghana
15	1	Pat40	*pf+poc* ^#3^	*poc* (70)			1 *poc* ^R^	Cameroon
16	1	Pat23	*pf+poc* ^#3^	*pv*^#^ (28)			1 *pv* ^F^	Congo
17	1	Pat32	*pf+pow* ^#3^	*poc* (137)			1 *poc* ^R^	Ghana
18	1	Pat41	*poc* ^#3^ *+pow* ^#3^	*poc* (91)			1 *poc* ^R^	Ghana
19	1	Pat37	*pf* ^#2^ *+pv*	*pv*^#3R^ (204)	*pv* (9)		1 *pv* ^R^*, 1 *pv* ^F^	Congo

pf, Plasmodium falciparum; pv, P. vivax; pm, P. malariae; poc, P. ovale curtisi; pow, P. ovale wallikeri, n, number of patients. Mixed infections are shown with “+,” e.g., pf+pv represents mixed infections of P. falciparum and P. vivax. The subscript # and letters refer to treatment protocols (#1, #2, and #3) and classifications of recurrence (F, R, D). F, treatment failure (8–42 days); R, relapse (pv or po, >42 days); D, parasite dormancy or present in low parasitemia (pf, >42 days). All patients involved those without traveling abroad from the first episode of illness to recurrence.

*Involved non-targeted treatment for relapse.

### Genetic Diversity and MOI of *P. falciparum* Determined by *pfmsp1* and *pfama1* Markers

A total of 49 and 33 P*. falciparum* samples were performed by deep sequencing of *pfmsp1* and *pfama1* markers, respectively. An average sequencing coverage depth for the major haplotypes was 11,136 and 8,656 for *pfmsp1* and *pfama1* markers, respectively (**Supplementary Table 3**). *pfmsp1* showed a total of 10 length polymorphic fragments (266, 284, 293, 302, 311, 320, 329, 347, 356, and 392 bp) in the 27 predominant haplotypes within samples (**Supplementary Figure 2**), whereas *pfama1* had an identical length of 390 bp in the 20 predominant haplotypes within samples (**Supplementary Table 7**). Seventy percent (42/60) of *pfmsp1* haplotypes were new haplotypes, whereas only 11.1% (3/27) of *pfama1* haplotypes were new haplotypes compared to the sequences available in GenBank. Among the 20 predominant haplotypes of *pfama1*, a total of 31 segregating sites were found with a nucleotide diversity Pi = 0.025 and a haplotype diversity Hd = 0.960. Overall, the number of clones ranged from 1 to 6 with a mean MOI of 2.2 ± 0.2, 95% CI [1.7, 2.6] determined for the 50 P*. falciparum* samples by combining the two markers. Of these, 44% (22/50) samples had multiclonal infections (**Supplementary Figure 3**). The *pfmsp1* and *pfama1* sequences of predominant haplotypes were deposited at GenBank with accession numbers MT947241–MT947300 and MT947313–MT947339.

### Genetic Diversity and MOI of *P. vivax* and *P. ovale* Determined by *msp1* and *ama1* Markers

**Figure 2:**
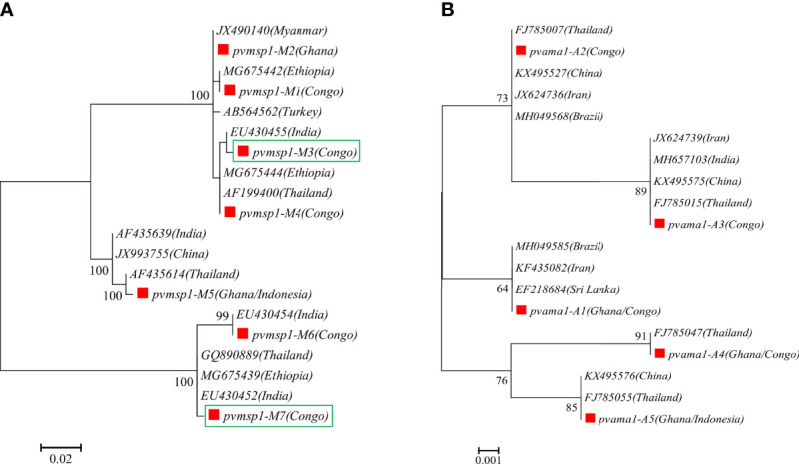
Molecular phylogenetic analysis by the maximum likelihood method based on haplotypes determined by amplicon deep sequencing in *P. vivax*. **(A)** *pvmsp1* phylogenetic tree for the seven haplotypes (M1–M7) and 13 sequences retrieved from GenBank (AB564562, AF199400, AF435614, AF435639, EU430452, EU430454, EU430455, GQ890889, JX490140, JX993755, MG675439, MG675442, MG675444). **(B)** *pvama1* phylogenetic tree for the five haplotypes (A1–A5) and 14 sequences retrieved from GenBank (MH049585, FJ785007, FJ785015, FJ785055, MH049568, FJ785047, EF218684, JX624736, JX624739, MH657103, KX495527, KX495575, KX495576, KF435082). *pf*, *P. falciparum*; *pv*, *P. vivax*; *pm*, *P. malariae*; *poc*, *P. ovale curtisi*; and *pow*, *P. ovale wallikeri.* Red square color mark represents haplotypes identified in this study. The new haplotypes are showing in green boxes.

**Figure 3:**
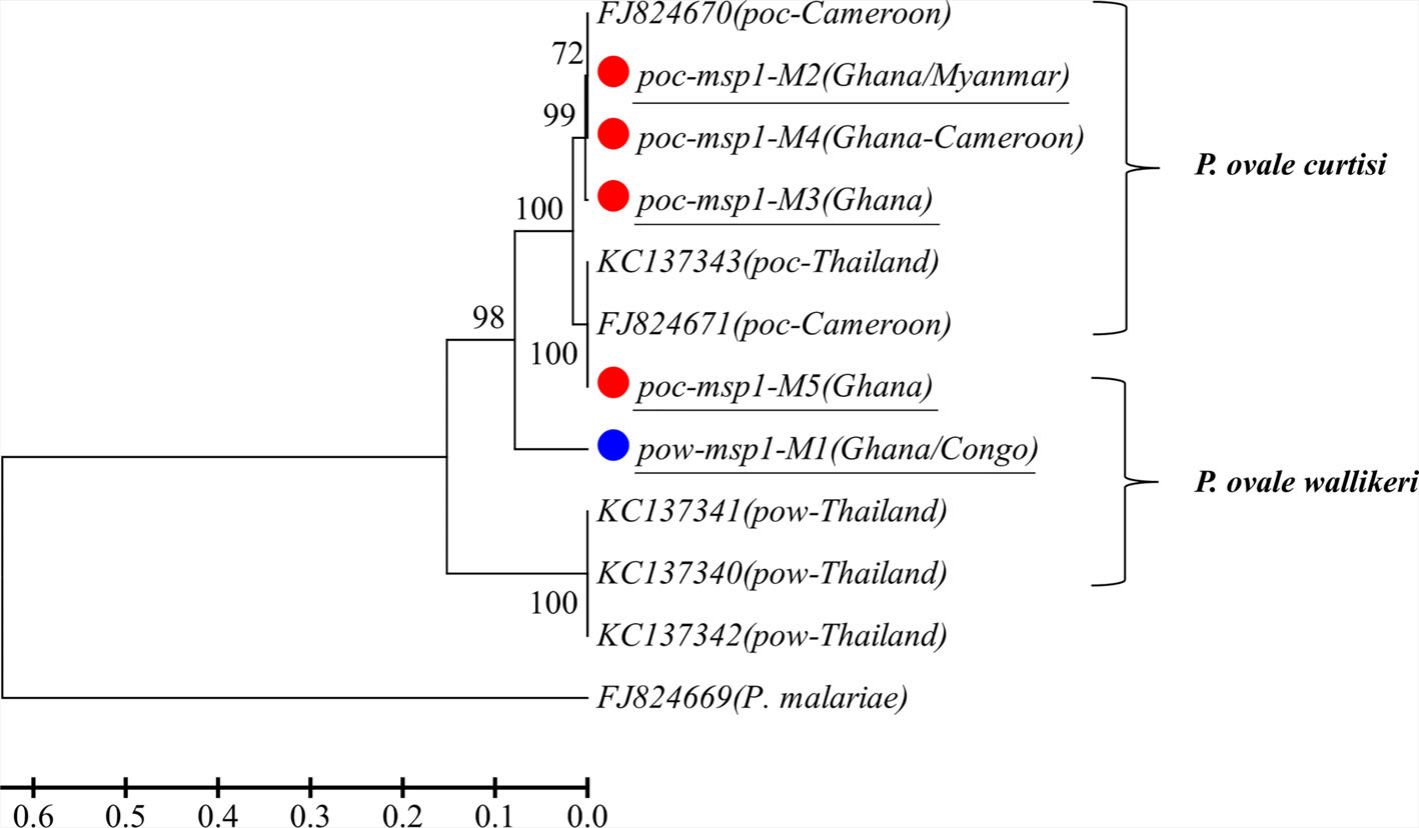
Phylogenetic tree analysis of *msp1* haplotypes (M1–M5) determined by amplicon deep sequencing in *P. ovale*. *poc*, *P. ovale curtisi*; *pow*, *P. ovale wallikeri. P. malariae* is used as outgroup. Color mark represents haplotypes identified in this study. The novel haplotypes show the underlined label.

A total of 14 and 15 P*. vivax* samples were performed for deep sequencing of *pvmsp1* and *pvama1* markers, respectively. An average sequencing coverage depth for the major haplotypes was 9,259 and 9,250 for *pvmsp1* and *pvama1* markers, respectively (**Supplementary Table 3**). No length polymorphism was found within samples in both *pvmsp1* amplicon (309 bp) and *pvama1* amplicon (288 bp). Seven haplotypes were identified for *pvmsp1* and 5 for *pvama1*. Two of the seven *pvmsp1* haplotypes were new, whereas none of the five *pvama1* haplotypes were new compared to those sequences in the GenBank (**Supplementary Table 7**). Molecular phylogenetic analysis indicated that most haplotypes were shared among Africa and Asia (**Figure 2**). Overall, MOI = 1 (single infection) was found for all the 15 P*. vivax* samples by combining the two markers. For *P. ovale*, a total of 14 samples were subjected to deep sequencing of the *pomsp1* marker. An average sequencing coverage depth for the major haplotypes was 10,759 joined reads (**Supplementary Table 3**). *pomsp1* showed a total of four length polymorphic fragments (289, 316, 331, and 352 bp) and five haplotypes (four for *poc* and one for *pow*). Four of the five *pomsp1* haplotypes were novel haplotypes compared to those sequences in GenBank. Phylogenetic tree analysis indicated that Ghana had all five haplotypes, whereas Congo, Ghana, and Myanmar had one haplotype for each country (**Figure 3**). The *pvmsp1*, *pvama1*, and *pomsp1* sequences of predominant haplotypes were deposited at NCBI GenBank with accession numbers MT947301–MT947312 and MT947340–MT947344.

### Comparison of Haplotypes Between First and Recurrent Malaria Episodes by *msp1* and *ama1* Markers

**Figure 4:**
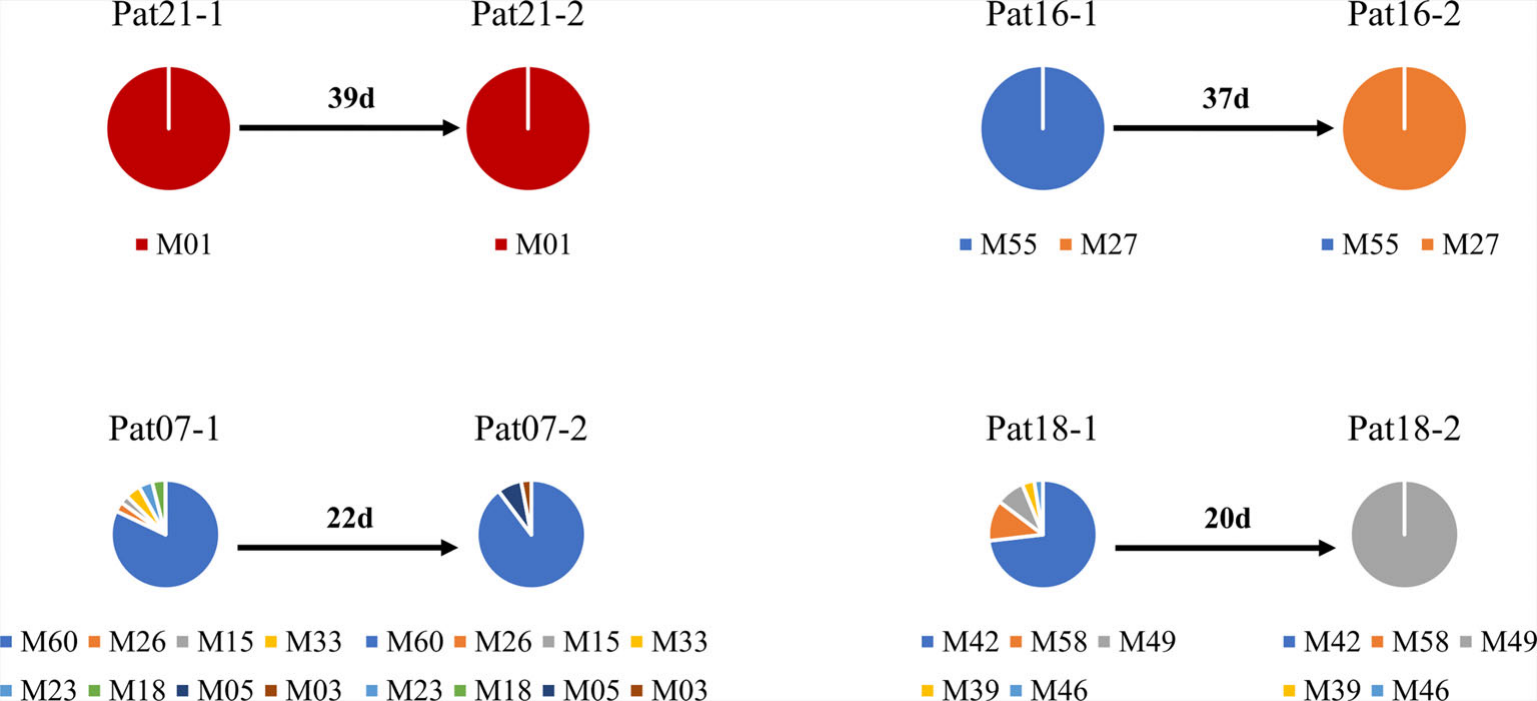
Pie chart shows the recurring patterns of *P. falciparum* by *pfmsp1* haplotypes (“M” with two-digit numbers). Malaria patient episodes are indicated above each pie chart; the number above the arrow indicates malaria recurrence intervals (days). Additional information is shown in **Supplementary Table 1**.

The deep sequencing of *pfmsp1* and *pfama1* on the first and second episodes was successfully performed for a total of 12 patients infected with *P. falciparum* (**Table 2**). Combining *pfmsp1* and *pfama1* haplotypes, three patients had identical genotypes and three patients had a reduced number of haplotypes from the first to the second episode, whereas two patients had partial changes of haplotype number at recurrence. One patient had an increased number of haplotypes, and three patients had changed haplotypes at recurrence. Overall, one patient failed treatment within 14 days. Nine patients had a second infection 15 to 39 days posttreatment and shared one or more haplotypes with the first episode. The remaining two patients showed different haplotypes between the first and second episodes and were considered as indeterminate. The pie chart showed some examples of detailed recurring patterns by the changes of *pfmsp1* haplotype frequencies (**Figure 4**).

**Table 2:** Comparisons of *pfmsp1* and *pfama1* combined haplotypes between first malaria episode and recurrent malaria.

Patient ID	Visited country	First malaria episodes (*pfmsp1* + *pfama1*)^†^	Second malaria episodes (*pfmsp1* + *pfama1*)	Interval (days)	Haplotype^‡^
Pat17	Ghana	M01 + A16	M01 + A16	24	Identical
Pat21	Ghana	M01 + A05	M01 + A05	39	Identical
Pat27	Ghana	M01 + NN	M01 + NN	15	Identical
Pat18	Ghana	M42M58M49M39M46 + A17A08A18	M49 + A08	20	Reduced
Pat04	Cameroon	M01M09 + NN	M01 + NN	15	Reduced
Pat26	Ghana	M09M31M11M30M29 + NN	M09 + NN	21	Reduced
Pat02	Ghana	M16M05 + A02	M16M08M05 + NN	22	Partially changed
Pat07	Congo	M60M26M33M23M15M18 + A05	M60M05M03 + A05	22	Reduced, changed
Pat20	Ghana	M51 + A08	M51M36M52M28 + NN	17	Increased
Pat12	Congo	M50 + A11	M17M10 + NN	13	Changed
Pat16	Ghana	M55 + A13A14	M27 + A15	37	Changed
Pat24	Ghana	M35M56M32 + A22A23A21	M07M05M31 + NN	20	Changed

“M” represents haplotype of pfmsp1, “A” represents haplotype of pfama1. “NN” represents missing data of haplotypes.

^†^Combined haplotypes of pfmsp1 and pfama1 markers.

^‡^“haplotype identical” means the same components of haplotypes in recurrent episodes; “haplotype reduced” means number of haplotypes decreased in recurrent episodes; “haplotype changed” means different haplotype (s) in recurrent episodes.

**Figure 5:**
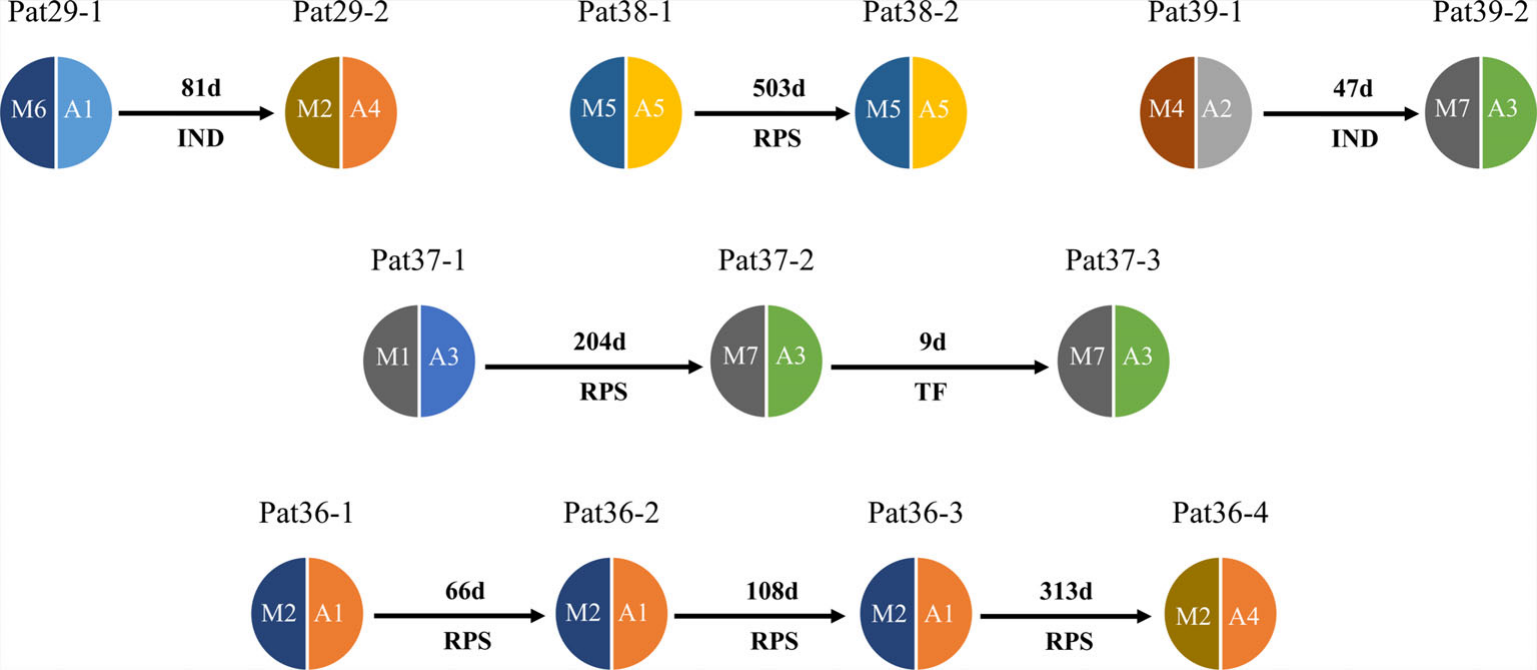
Pie chart shows haplotype changes between first episode and recurrence of *P. vivax.* Malaria patient episodes are indicated above each pie chart; arrows indicate recurrence interval (days); M1–M7 represent *pvmsp1* haplotypes, A1–A5 represent *pvmasp1* haplotypes. RPS = relapse (defined by shared one or more haplotypes after 42 days post treatment), TF = treatment failure (within 42 days), IND, indeterminate (no shared haplotype). Additional information is shown in **Supplementary Table 1**.

The deep sequencing of *pvmsp1* and *pvama1* on the first and recurrent episodes was successfully performed for a total of five patients infected with *P. vivax*. Of these, three patients underwent two rounds of treatments, whereas one patient had three rounds of treatments and one had four rounds of treatments. All-round of treatments were the same prescriptions (**Supplementary_Methods**). Except Pat37 who had an early treatment failure after the second episode, all patients had a recurrent infection from 47 to 503 days in which four had identical combined *pvmsp1* and *pvama1* haplotypes, while four had different haplotypes between two episodes (**Figure 5**). Although Pat38 had additional travel history to Ghana, the haplotype (M5+A5) of the recurrent episode was identical to the first episode and different from Ghana as identified in this study. Overall, all the five *P. vivax* recurrences except the one as treatment failure could probably be considered relapses since these patients did not travel after the first episode, and there is no local malaria transmission. For *P. ovale*, there was one patient (Pat42) identified by *pomsp1* with identical genotypes between second episode and third episode with a time length interval of 24 days, whereas the other patients had only one episode successfully genotyped.

### Mutations of Antimalarial Drug Resistance Genes in *P. falciparum* Infections

A total of eight patients were successfully genotyped for mutations in the five antimalaria drug resistance genes (**Supplementary Table 8**). All the patients had recurrence and travel history before the first malaria attack from Ghana, except one (Pat07) from Congo. However, Pat18 had malaria recrudesces and showed reduced genotypes 20 days after treatment, suggesting the role of parasite-resistant genotypes. There was no statistically significant difference between the first episode and recurrent infections in the mutation frequencies of all the antimalaria drug resistance genes (Fisher’s exact test, *p* > 0.05). The sample size for patients with recurrence may not be enough to exclude its association with mutations.

### Profiles of CYP2D6 Gene in *P. vivax* Patients

A total of four patients were successfully genotyped for mutations of the CYP2D6 gene in *P. vivax* patients. The results indicated that those patients’ CYP2D6 genotypes correspond to *4N/*2A, *2A/*4N, *2A/*36, and *4N/*15 allele variants, respectively (**Supplementary Table 1**). The overall genotype activity scores were 1, 1, 1, and 0, respectively. The CYP2D6 genotype activity scores ≤ 1.0 were considered poor primaquine metabolizers in *P. vivax* infection (Baird et al., 2018).

## Discussion

Imported malaria cases to areas free of malaria transmission provide a unique opportunity to investigate patterns of malaria reappearance. Next-generation targeted amplicon deep-sequencing approaches empower a high-throughput, quantitative method for identification of *Plasmodium* species and mixed-species infections as well as detection of multiclonal infections, including minor alleles present within a sample (Koepfli and Mueller, 2017; Lalremruata et al., 2017; Lerch et al., 2017; Zhong et al., 2018). In the study, mixed-species infections were detected in 15 out of the 93 malaria episodes (~16%). Most of these were *P. ovale curtisi* coinfected with *P. falciparum.* This result is consistent with the previous study in parasite samples from Thailand and West Africa (Saralamba et al., 2019). A high proportion of mixed-species infections was also reported in other studies using ADS, in which up to quadruple mixed-species infections were observed (Lalremruata et al., 2017). Compared to CYTB, small-subunit ribosomal RNA (18S rRNA) had much higher polymorphism and multiple different haplotypes or multiple copies (up to 5) within the sample as seen in this study. Although both markers can be used to discriminate *P. ovale* subspecies, no mixed-species infection was detected by the CYTB marker when up to 15 mixed-species infections were found by using the SSU marker. Likewise, this SSU marker should be a good candidate and well-suited molecular target for the detection and discrimination of all *Plasmodium* species. Compared to *ama1*, *msp1* had a higher polymorphism similar to our previous study in Kenya samples (Zhong et al., 2018), which is also a good candidate marker for amplicon deep sequencing to detect multiclonal infections.

Although *P. falciparum* infections were responsible for the majority of imported malaria cases in China, there was a large proportion (25.9% for *P. ovale* and 19.4% for *P. vivax* including mixed species) of non-falciparum infections cases in this study. Recently, *P. ovale* infections have become more common in Africa, suggesting that *P. ovale* is becoming an increasingly important malaria parasite, highlighting the need for attention toward non-falciparum malaria (Yman et al., 2019; Zhou et al., 2019; Lin et al., 2020). A large proportion of *P. ovale* infections and mixed-species infections were also evidenced in the imported malaria cases from Africa to China as seen in the current study. There is very limited genomic information of *P. ovale* available in the NCBI sequence database. In this study, we found a total of seven haplotypes (four for *P. ovale curtisi* and three for *P. ovale wallikeri*) by the SSU marker, five haplotypes (four for *P. ovale curtisi* and one for *P. ovale wallikeri*) from the *pomsp1* marker. Among these, four out of the five *pomsp1* haplotypes were identified as novel haplotypes (all from *P. ovale curtisi*); its potential role in antimalaria resistance needs to be studied further. The high proportion of *P. vivax* infections and relapse was observed in the study, possibly due to positive Duffy antigens elevating susceptibility to vivax malaria in Asian patients (Miller et al., 1976; de Carvalho and de Carvalho, 2011; Howes et al., 2011).

We found a very complex pattern of malaria reappearance in these imported malaria cases by examining *msp1* and *ama1* markers. For *P. falciparum*, the same genotypes were detected from 15 to 39 days after ACT, while a reduced genotype was observed from 15 to 21 days after ACT. Starting from 17 days, an increased number of genotypes were observed in Patient 20, whereas totally different genotypes were found from 13 to 37 days after ACT. These complex patterns of reappearance may be an indication of early treatment failures (Borrmann et al., 2003; Jackson et al., 2006; Huang et al., 2012; Madamet et al., 2017), antimalarial drug resistance (Lu et al., 2017), late treatment failures (Borrmann et al., 2003; Sondén et al., 2016), and ring-stage parasites associated with artemisinin resistance (Zhang et al., 2017). However, there was no resistance-associated *pfk13* mutation detected except for N-terminal insertion (NN) and K189T/N; their roles in resistance are not yet determined (Group, 2019; Zhao et al., 2019). The changed allele observed in reappearance might be explained by early detection failure due to the presence of minor alleles in multiclonal infections, or parasites remaining dormant and relapsing during pregnancy (Theunissen et al., 2009; Malvy et al., 2018). Some of the tested samples had *P. falciparum* drug-resistance alleles to chloroquine with the changed multiclonal infections detected, further supporting the presence of resistant parasites below the limit of detection. For *P. vivax*, a long-latency period of 503 days between first episode and relapse was observed with the same combined haplotypes from Indonesia (Pat38), although this patient had an additional trip to Ghana. The short or long latency relapses after primaquine treatment for *P. vivax* infections could be explained by parasite drug resistance (Baird, 2004) or patients with a poor metabolizer CYP2D6 variant for three-fourths of vivax cases, as detected in the current study. There is little information about drug resistance in *P. ovale*; further study is needed to investigate the association between genetic and drug resistance in this species.

## Conclusion

This study elucidated patterns of malaria reappearance, especially for the less studied non-falciparum malaria parasite species. A high proportion of *P. vivax* and *P. ovale* parasites carriers, as well as a high proportion of mixed-species infections, may have important implications for non-falciparum malaria control and treatment strategies. The high recurrence rate and unexpectedly complex patterns of imported malaria from Africa to non-endemic countries pose a risk for increased local transmission and might undermine malaria control and elimination. Appropriate interventions and monitoring and accurate diagnostics using molecular methods are essential for imported malaria management. Finally, the study highlighted the power of ADS applications in molecular epidemiological studies of imported malaria.

## Data Availability Statement

The datasets presented in this study can be found in online repositories. The names of the repository/repositories and accession number(s) can be found in the article/**Supplementary Material**.

## Ethics Statement

The studies involving human participants were reviewed and approved by the Ethical Review Committee of Shanglin People’s Hospital, Guangxi, China. The patients/participants provided their written informed consent to participate in this study.

## Author Contributions

GY, ZY, and DZ conceived and designed the study. XH wrote the first draft of the paper. DZ and LC helped in performing data analysis and drafted the manuscript. XH, CZ, LP, LZ, YQ, MP, SW, WZ, ZX, XC, YW, YS, and YH conducted the sample collection and experiments. All authors contributed to the article and approved the submitted version.

## Funding

This work was supported by grants (31860604 and U1802286) from the National Natural Science Foundation of China and by a grant (2018ZF0081) from Major Science and Technology Projects of Yunnan and Technology Cooperation-Yunnan International Science and Technology Cooperation Base (202003AE140004). LC was supported by a grant U19AI089672 from the National Institutes of Health, USA. GY was supported by the National Institutes of Health USA (U19AI129326). CZ was funded by a grant from the Youth Fund Project of People’s Hospital of Guangxi Zhuang Autonomous Region, China (QN2017-10). MP was funded by a grant (ZC20153012) from the Science and Technology Bureau Programs of Nanning, Guangxi, China. YQ was funded by the Scientific Research Project of Health Committee of Guangxi Zhuang Autonomous Region (20191525). WZ was supported by the Education Department Fund of Yunnan Province (2019J1184). XC and ZX were sponsored by the Yunnan Applied Basic Research Projects-Union Foundation (2018FE001-190 and 2019FE001-015, respectively). YW was supported by the Hundred-Talent Program of Kunming Medical University (60117190439), and the Foundation of the Education Department of Yunnan Province (2018JS151) and the Creative Experimental Project of Kunming Medical University and Yunnan Province.

## Conflict of Interest

The authors declare that the research was conducted in the absence of any commercial or financial relationships that could be construed as a potential conflict of interest.

## Publisher’s Note

All claims expressed in this article are solely those of the authors and do not necessarily represent those of their affiliated organizations, or those of the publisher, the editors and the reviewers. Any product that may be evaluated in this article, or claim that may be made by its manufacturer, is not guaranteed or endorsed by the publisher.
