# Supplementary material for: Unraveling the Complexity of Imported Malaria Infections by Amplicon Deep Sequencing
Source: Front Cell Infect Microbiol. 2021 Sep 14;11:725859. doi: 10.3389/fcimb.2021.725859 (PMC8477663; doi:10.3389/fcimb.2021.725859)
Supplement: Supplementary file 1 [file DataSheet_1.pdf]

Supplemental Methods: Protocols for treatment of imported malaria in Shanglin county, Guangxi, China.

Malaria patients were hospitalized in Shanglin county and treated following the guidelines of Chinese Center for Disease Control and Prevention (CDC) and WHO recommendations on the diagnosis and treatment of uncomplicated and severe malaria. For those *P. falciparum* patients, all were treated with intravenous (IV) injections of artesunate and co-treated with oral dihydroartemisinin-piperaquine (DHP) for 3 days (Protocol #1) or 8 days course (Protocol #2) after hospitalized. For *P. vivax* and *P. ovale* patients, 8-day course of primaquine (PQ) regimen for 8 days were prescribed (Protocol #3). All *P. falciparum* patients were treated with primaquine 0.75 mg/kg as a single dose on the 3rd day at the time of discharge from hospital.

Protocol #1: Artesunate, Guilin Pharmaceutical (Shanghai) Co. LTD (Approval date: 02/12/2015).

Day0:120mg (or 2.4mg/kg);12h:120mg (or 2.4mg/kg);
Day1:120mg (or 2.4mg/kg);
Day2:120mg (or 2.4mg/kg).
Maximum Dosage:480mg.
120mg once per day after patient is tolerated to oral medication.

Protocol #2: Artesunate, NHFPCC (Recommendation date: 5/20/2016).

Day0:120mg (or 2.4mg/kg);12h:120mg (or 2.4mg/kg);
Day1:120mg (or 2.4mg/kg);
Day2:120mg (or 2.4mg/kg);
Day3:120mg (or 2.4mg/kg);
Day4:120mg (or 2.4mg/kg);
Day5:120mg (or 2.4mg/kg);
Day6:120mg (or 2.4mg/kg).
Maximum Dosage:960mg.
120mg once per day after symptom relief or patient is tolerated to oral medication.

Protocol #3: To improve adherence, China recommends a shorter 8-day course of primaquine (PQ) regimen (0.375 mg/kg/day; total dose of 3.0 mg/kg) for relapsing malaria. Treatment included 3 days of intravenous artesunate (420 mg total), and 3 days of chloroquine (1550 mg total), and 8 days of primaquine (22.5g/day,180 mg total).

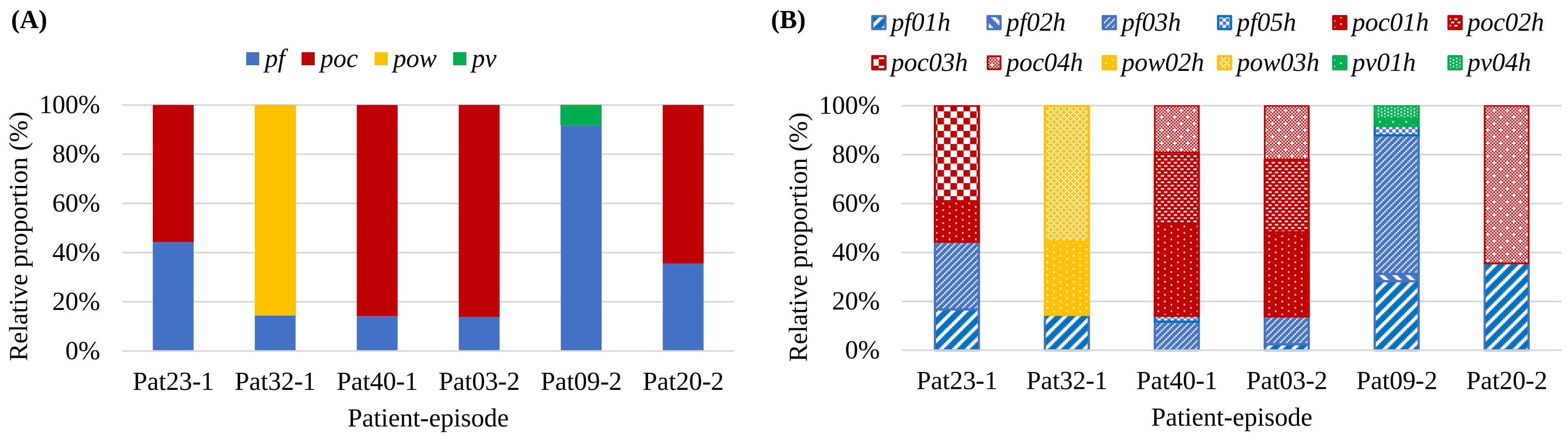

Supplementary Figure 1 Relative proportion of mixed-species infections determined by amplicon deep sequencing of SSU marker. (**A**) relative proportion of the 4 *Plasmodium* species (*pf*, *poc*, *pow*, and *pv*) in first episodes of 3 patients (Pat23, Pat32, and Pat40) and second episodes of 3 patients (Pat03, Pat09, and pat20); (**B**) relative proportion of the 12 haplotypes (*pf*: 01h,02h, 03h, and 05h; *poc*: 01h, 02h, 03h, and 04h; *pow*: 02h and 03h; *pv*: 01h and 04h) in first episodes of 3 patients (Pat23, Pat32, and Pat40) and second episodes of 3 patients (Pat03, Pat09, and pat20). *pf*, *Plasmodium falciparum; pv, P. vivax; pm, P. malariae; poc, P. ovale curtisi;* and *pow, P. ovale wallikeri*.

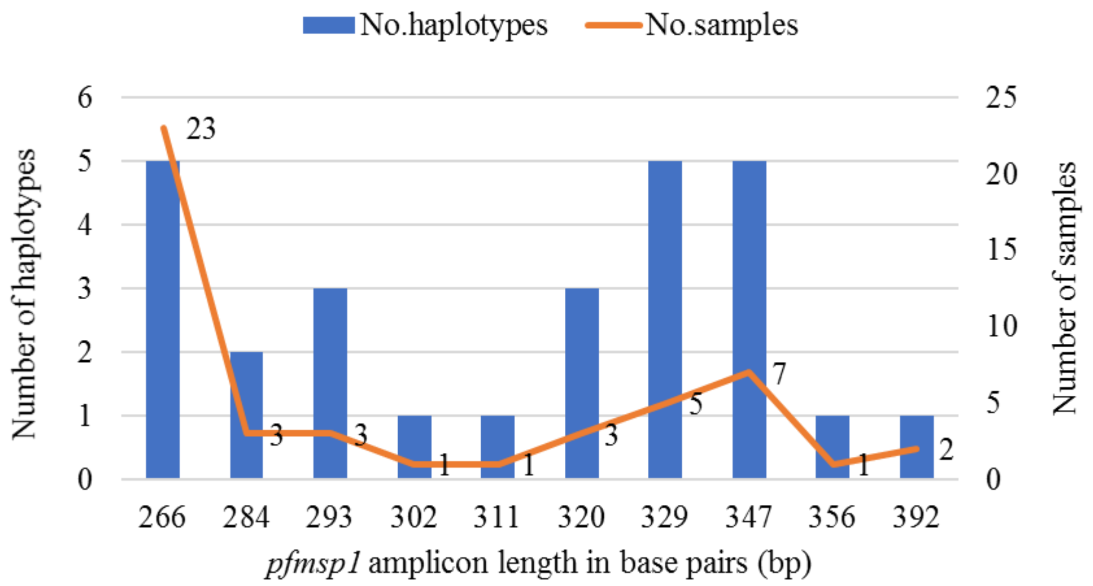

Supplementary Figure 2 Histogram distribution of *pfmsp1* amplicon length (bp) and number of predominant haplotypes in the imported malaria infection of *Plasmodium falciparum*.

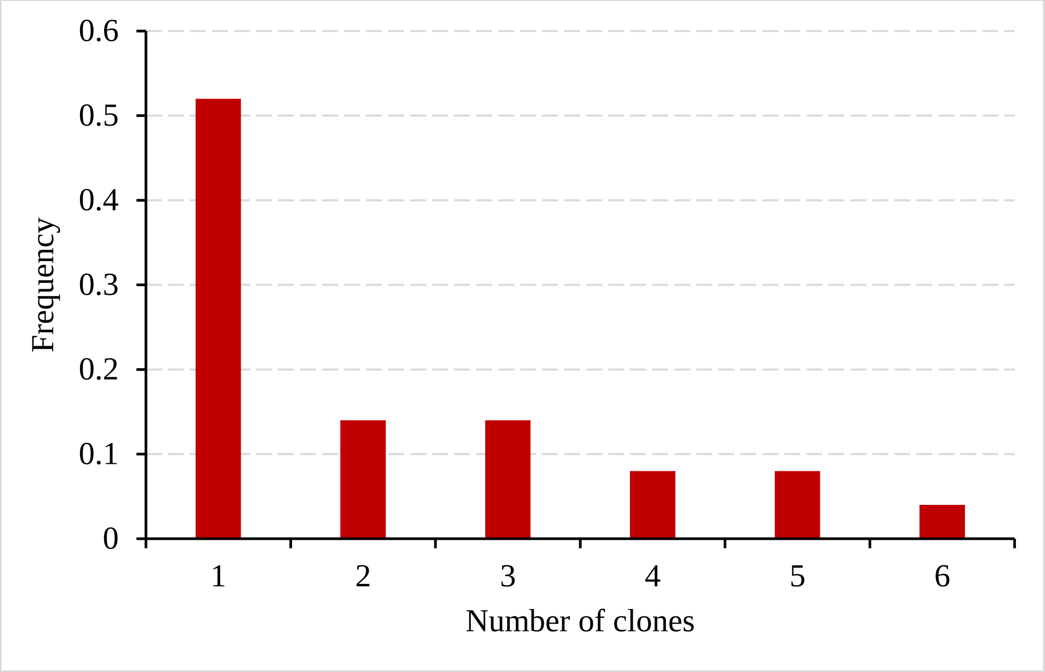

Supplementary Figure 3 Frequency distribution of number of clones determined by *pfmsp1* and *pfama1* genes in the imported malaria infection of *Plasmodium falciparum.*

Table S1 information data sheet of imported malaria cases used in the study.

Sample	Patient	Episode of illness	Year	Travel Country	Diagnosis date	Days between episodes
1	Pat01	1	2016	Ghana	Jan-16	
2	Pat01	2	2016		Feb-16	21
3	Pat02	1	2016	Ghana	Feb-16	
4	Pat02	2	2016		Feb-16	22
5	Pat02	3	2017	Ghana	Feb-17	366
6	Pat03	1	2016	Cameroon	Feb-16	
7	Pat03	2	2016		Feb-16	17
8	Pat04	1	2016	Cameroon	Mar-16	
9	Pat04	2	2016		Apr-16	15
10	Pat05	1	2016	Cameroon	Mar-16	
11	Pat05	2	2018	Congo	Feb-18	696
12	Pat06	1	2016	Ghana	Apr-16	
13	Pat06	2	2017	Ghana	Aug-17	461
14	Pat07	1	2016	Congo	May-16	
15	Pat07	2	2016		Jun-16	22
16	Pat08	1	2016	Cameroon	Jun-16	
17	Pat08	2	2016		Aug-16	71
18	Pat09	1	2017	Ghana	Feb-17	
19	Pat09	2	2017		Feb-17	3
20	Pat10	1	2016	Ghana	Dec-16	
21	Pat10	2	2016		Dec-16	4
22	Pat11	1	2016	Congo	Sep-16	
23	Pat11	2	2016		Dec-16	97
24	Pat12	1	2017	Congo	Aug-17	
25	Pat12	2	2017		Aug-17	13
26	Pat13	1	2017	Myanmar	Nov-17	
27	Pat13	2	2017		Nov-17	6
28	Pat14	1	2017	Cameroon	Nov-17	
29	Pat14	2	2017		Dec-17	36
30	Pat15	1	2017	Ghana	Dec-17	
31	Pat15	2	2017		Dec-17	22
32	Pat16	1	2017	Ghana	Dec-17	
33	Pat16	2	2018		Jan-18	37
34	Pat17	1	2018	Ghana	Feb-18	
35	Pat17	2	2018		Mar-18	24
36	Pat18	1	2017	Ghana	Apr-17	
37	Pat18	2	2017		May-17	20
38	Pat19	1	2017	Ghana	Apr-17	
39	Pat19	2	2017		Jul-17	83
40	Pat20	1	2017	Ghana	Apr-17	
41	Pat20	2	2017		May-17	17
42	Pat21	1	2017	Ghana	May-17	
43	Pat21	2	2017		Jun-17	39
44	Pat22	1	2017	Ghana	May-17	
45	Pat22	2	2017		Jun-17	18
46	Pat23	1	2017	Congo	May-17	
47	Pat23	2	2017		Jun-17	28
48	Pat24	1	2017	Ghana	Jun-17	
49	Pat24	2	2017		Jul-17	20

Sample	Patient	Episode of illness	Year	Travel Country	Diagnosis date	Days between episodes
50	Pat25	1	2017	Ghana	Jul-17	
51	Pat25	2	2017		Sep-17	58
52	Pat26	1	2017	Ghana	Jul-17	
53	Pat26	2	2017		Aug-17	21
54	Pat27	1	2017	Ghana	Jul-17	
55	Pat27	2	2017		Aug-17	15
56	Pat28	1	2016	Ghana	Sep-16	
57	Pat28	2	2017	Ghana	Nov-17	401
58	Pat29	1	2016	Congo	Oct-16	
59	Pat29	2	2017		Jan-17	81
60	Pat30	1	2016	Ghana	Nov-16	
61	Pat30	2	2016		Dec-16	31
62	Pat31	1	2017	Ghana	Apr-17	
63	Pat31	2	2017		Dec-17	250
64	Pat32	1	2017	Ghana	Jan-17	
65	Pat32	2	2017		May-17	137
66	Pat33	1	2017	Congo	Jan-17	
67	Pat33	2	2017		Jan-17	12
68	Pat34	1	2017	Ghana	Jan-17	
69	Pat34	2	2017		Apr-17	103
70	Pat35	1	2017	Ghana	Jan-17	
71	Pat35	2	2017		May-17	125
72	Pat36	1	2016	Ghana	Dec-16	
73	Pat36	2	2017		Mar-17	66
74	Pat36	3	2017		Jun-17	108
75	Pat36	4	2018		Apr-18	313
76	Pat37	1	2016	Congo	Nov-16	
77	Pat37	2	2017		Jun-17	204
78	Pat37	3	2017		Jun-17	9
79	Pat38	1	2016	Indonesia	Mar-16	
80	Pat38	2	2017	Ghana	Aug-17	503
81	Pat39	1	2017	Congo	Mar-17	
82	Pat39	2	2017		May-17	47
83	Pat40	1	2017	Cameroon	Jun-17	
84	Pat40	2	2017		Aug-17	70
85	Pat41	1	2017	Ghana	Jul-17	
86	Pat41	2	2017		Oct-17	91
87	Pat42	1	2017	Ghana	Jul-17	
88	Pat42	2	2018		Feb-18	236
89	Pat42	3	2018		Mar-18	24
90	Pat43	1	2016	Cameroon	Dec-16	
91	Pat43	2	2017	Ghana	May-17	167
92	Pat44	1	2017	Congo	Jun-17	
93	Pat44	2	2017		Aug-17	74

Sample	Treatment	Age range	Gender	Occupation	Final_ID	Nested PCR	CYTB
1	Protocol #1	60-69	Male	Gold miner	pf	pf	pf
2	Protocol #1	60-69			pf	pf	pf
3	Protocol #1	40-49	Male	Gold miner	pf	pf	pf
4	Protocol #1	40-49			pf	pf	pf
5	Protocol #2	40-49			pf	pf	pf
6	Protocol #1	50-59	Male	Gold miner	pf	pf	
7	Protocol #3	50-59			pf+poc	po	poc
8	Protocol #1	20-29	Male	Gold miner	pf	pf	pf
9	Protocol #1	20-29			pf	pf	pf
10	Protocol #3	40-49	Male	Gold miner	poc	po	poc
11	Protocol #2	40-49			pf	pf	
12	Protocol #1	40-49	Male	Gold miner	pf	pf	
13	Protocol #3	40-49			poc	po	poc
14	Protocol #1	40-49	Male	Gold miner	pf	pf	pf
15	Protocol #2	40-49			pf	pf	pf
16	Protocol #2	40-49	Male	Gold miner	pf	pf	pf
17	Protocol #3	40-49			poc	po	poc
18	Protocol #2	30-39	Male	Gold miner	pf	pf	pf
19	Protocol #3	30-39			pf+pv	pf+pv	pf+pv
20	Protocol #2	30-39	Male	Gold miner	pf	pf	pf
21	Protocol #2	30-39			pf	pf	pf
22	Protocol #2	40-49	Male	Gold miner	pf	pf	pf
23	Protocol #2	40-49			pf	pf	
24	Protocol #2	50-59	Male	Gold miner	pf	pf	pf
25	Protocol #2	50-59			pf	pf	pf
26	Protocol #2	20-29	Male	Visitor	pf+pm	pf	pm
27	Protocol #3	20-29			pf+poc	pf+po	pf
28	Protocol #2	30-39	Male	Gold miner	pf	pf	pf
29	Protocol #3	30-39			pm	pm	pm
30	Protocol #2	20-29	Male	Gold miner	pf	pf	
31	Protocol #2	20-29			pf	pf	
32	Protocol #2	30-39	Male	Gold miner	pf	pf	pf
33	Protocol #2	30-39			pf	pf	pf
34	Protocol #2	30-39	Male	Gold miner	pf	pf	pf
35	Protocol #2	30-39			pf	pf	pf
36	Protocol #2	40-49	Male	Gold miner	pf	pf	pf
37	Protocol #2	40-49			pf	pf	pf
38	Protocol #2	30-39	Male	Gold miner	pf	pf	pf
39	Protocol #3	30-39			poc	po	poc
40	Protocol #2	20-29	Male	Gold miner	pf	pf	pf
41	Protocol #2	20-29			pf+poc	pf	pf
42	Protocol #2	20-29	Male	Gold miner	pf	pf	pf
43	Protocol #2	20-29			pf	pf	pf
44	Protocol #3	40-49	Male	Gold miner	poc	po	poc
45	Protocol #2	40-49			pf	pf	pf
46	Protocol #3	50-59	Male	Gold miner	pf+poc	po	
47	Protocol #3	50-59			pv	pv	pv
48	Protocol #2	30-39	Male	Gold miner	pf	pf	pf
49	Protocol #2	30-39			pf	pf	pf

Sample	Treatment	Age range	Gender	Occupation	Final_ID	Nested PCR	CYTB
50	Protocol #2	40-49	Male	Gold miner	pf	pf	pf
51	Protocol #2	40-49			pf	pf	
52	Protocol #2	50-59	Male	Gold miner	pf+poc	pf	poc
53	Protocol #2	50-59			pf+poc	pf	
54	Protocol #2	40-49	Male	Gold miner	pf	pf	pf
55	Protocol #3	40-49			pf+poc	po	poc
56	Protocol #2	20-29	Male	Gold miner	pf	pf	pf
57	Protocol #2	30-39			pf	pf	
58	Protocol #3	30-39	Male	Gold miner	pv	pv	pv
59	Protocol #3	30-39			pv	pv	
60	Protocol #2	50-59	Male	Gold miner	pf	pf	pf
61	Protocol #3	50-59			pv+poc	po	pv
62	Protocol #3	30-39	Male	Gold miner	pv	pv	pv
63	Protocol #3	30-39			pow	po	pow
64	Protocol #3	40-49	Male	Gold miner	pf+pow	po	
65	Protocol #3	40-49			poc	po	poc
66	Protocol #2	20-29	Male	Gold miner	pf	pf	pf
67	Protocol #2	20-29			pf	pf	pf
68	Protocol #2	40-49	Male	Gold miner	pf	pf	pf
69	Protocol #3	40-49			poc	po	poc
70	Protocol #2	20-29	Male	Gold miner	pf	pf	pf
71	Protocol #3	20-29			pv	pv	pv
72	Protocol #3	40-49	Male	Gold miner	pv	pv	pv
73	Protocol #3	40-49			pv	pv	pv
74	Protocol #3	40-49			pf+pv	pv	pv
75	Protocol #3	50-59			pv	pv	pv
76	Protocol #2	50-59	Male	Gold miner	pf+pv	pf+pv	pv
77	Protocol #3	50-59			pv	pv	pv
78	Protocol #3	50-59			pv	pv	pv
79	Protocol #3	40-49	Male	Visitor	pv	pv	pv
80	Protocol #3	50-59			pv	pv	pv
81	Protocol #3	40-49	Male	Gold miner	pv	pv	pv
82	Protocol #3	40-49			pv	pv	pv
83	Protocol #3	30-39	Male	Gold miner	pf+poc	po	poc
84	Protocol #3	30-39			poc	po	poc
85	Protocol #3	20-29	Male	Gold miner	poc+pow	po	pow
86	Protocol #3	20-29			poc	po	poc
87	Protocol #2	50-59	Male	Gold miner	pf	pf	pf
88	Protocol #3	50-59			pow	po	pow
89	Protocol #3	50-59			pow	po	pow
90	Protocol #2	20-29	Male	Gold miner	pf	pf	pf
91	Protocol #2	20-29			pf	pf	pf
92	Protocol #2	30-39	Male	Gold miner	pf	pf	pf
93	Protocol #3	30-39			pow	po	pow

Sample	SSU haplotype	pfmsp1 haplotype	pfmsp1-MOI	pfama1 haplotype
1	pf03h/pf01h			
2	pf03h/pf01h/pf05h/pf02h	M01	1	A01
3	pf03h/pf01h	M16M05	2	A02
4	pf03h/pf01h	M16M08M05	3	na
5	pf03h/pf01h	M25M14M22M38M05	5	A03
6	pf03h/pf01h			
7	poc01h/poc02h/poc04h/pf03h/pf01h			
8	pf03h/pf01h	M01M09	2	na
9	pf03h/pf01h	M01	1	na
10	poc03h/4/poc04h			
11		M05	1	na
12	pf03h/pf01h	M47	1	A12
13	poc01h			
14	pf03h/pf01h/pf05h	M60M26M33M23M15M18	6	A05
15	pf03h/pf01h	M60M05M03	3	A05
16	pf03h/pf01h	M34M02M05	3	na
17	poc04h/poc01h			
18	pf03h/pf01h	M48	1	A06
19	pf03h/pf01h/pv04h	M48	1	A06
20	pf03h/pf01h	M12M37M43M54M05	5	A07A02
21	pf03h/pf01h/pf05h/pf02h	M12M37M43M54	4	A07A02
22	pf03h/pf01h/pf02h	M01M59M27	3	A08A06A09A10
23				
24	pf03h/pf01h/pf05h/pf02h	M50	1	A11
25		M17M10	2	na
26	pm01h	M05	1	na
27	poc03h/poc01h	M21M09M13M04M52M05	6	na
28	pf03h/pf01h/pf05h/pf02h	M36M52M28	3	A12
29	pm01h			
30	pf03h/pf01h	M05	1	na
31				
32	pf03h/pf01h/pf05h/pf02h	M55	1	A13A14
33	pf03h	M27	1	A15
34	pf03h	M01	1	A16
35	pf03h/pf01h	M01	1	A16
36	pf03h/pf01h/pf02h/pf05h	M42M58M49M39M46	5	A17A08A18
37	pf03h/pf01h	M49	1	A08
38	pf03h	M41	1	A19
39	poc03h/poc01h			
40	pf03h	M51	1	A08
41	poc04h/pf01h	M51M36M52M28	4	na
42	pf03h	M01	1	A05
43	pf03h/pf01h	M01	1	A05
44	poc03h/4/poc01h			
45	pf03h/pf01h	M01	1	A20
46	poc03h/pf03h			
47	pv03h/pv01h/pv01h			
48	pf03h/pf01h	M35M56M32	3	A22A23A21
49		M07M05M31	3	na

Sample	SSU haplotype	pfmsp1 haplotype	pfmsp1-MOI	pfama1 haplotype
50	pf01h	M05M21	2	na
51	pf03h/pf01h	na		na
52	poc03h/poc01h	M09M31M11M30M29	5	na
53	poc03h/poc01h	M09	1	na
54	pf03h/pf01h	M01	1	na
55	poc04h/3/poc01h	M01	1	na
56	pf03h/pf01h/pf05h	M20	1	A24
57				
58	pv03h/pv04h/pv01h			
59				
60	pf03h/pf01h	M44M05	2	A06
61	poc04h/3/poc01h			
62	pv03h/pv01h	na		na
63	pow01h/pow03h			
64	pow03h/pow02h/pf01h			
65	poc01h			
66	pf03h/pf01h	M01	1	A25
67	pf03h/pf01h			
68	pf03h/pf01h	M01M24	2	A13A26A14
69	poc04h/poc01h			
70	pf03h/pf02h/pf01h	M19	1	A20A27
71				
72	pv04h/pv03h			
73	pv04h/pv03h/pv01h/pv02h			
74	pf03h			
75	pv03h/pv04h/pv01h			
76	pv03h/pv04h/pv01h	M05	1	A25
77	pv03h/pv01h			
78	pv03h/pv01h			
79	pv04h/pv01h			
80	pv04h/pv01h/pv02h			
81	pv03h/pv04h/pv01h			
82	pv03h/pv01h/pv02h/pv04h			
83	poc01h/poc02h/poc04h/pf03h/			
84	poc01h/poc02h/poc04h			
85	poc04h/3/poc01h			
86	poc01h/poc04h			
87	pf03h/pf01h/pf05h	M40M53M57M06	4	na
88	pow01h/pow03h			
89	pow01h/pow03h			
90	pf03h/pf01h			
91	pf04h/pf01h	M45	1	A26
92	pf03h/pf01h	na		A05
93	pow01h/pow03h			

Sample	pfama1-MOI	pfmsp1+ama1-MOI	pvmsp1 haplotype	pvama1 haplotype	pomsp1 haplotype
1					
2	1	1			
3	1	2			
4		3			
5	1	5			
6					
7					
8		2			
9		1			
10					M4
11		1			
12	1	1			
13					M4
14	1	6			
15	1	3			
16		3			
17					M4
18	1	1			
19	1	1	M5	A5	
20	2	5			
21	2	4			
22	4	4			
23					
24	1	1			
25		2			
26		1			
27		6			M2
28	1	3			
29					
30		1			
31					
32	2	2			
33	1	1			
34	1	1			
35	1	1			
36	3	5			
37	1	1			
38	1	1			
39					M2
40	1	1			
41		4			
42	1	1			
43	1	1			
44					M2
45	1	1			
46					
47			M3	A1	
48	3	3			
49		3			

Sample	pfama1-MOI	pfmsp1+ama1-MOI	pvmsp1 haplotype	pvama1 haplotype	pomsp1 haplotype
50		2			
51					
52		5			
53		1			
54		1			
55		1			
56	1	1			
57					
58			M6	A1	
59				A4	
60	1	2			
61					M5
62					
63					M1
64					
65					
66	1	1			
67					
68	3	3			
69					M3
70	2	2			
71					
72			M2	A1	
73			M2	A1	
74			M2	A1	
75			M2	A4	
76	1	1	M1	A3	
77			M7	A3	
78			M7	A3	
79			M5	A5	
80			M5	A5	
81			M4	A2	
82			M7	A3	
83					
84					M4
85					M4
86					
87		4			
88					M1
89					M1
90					
91	1	1			
92	1	1			
93					M1

Sample	pfK13_NN insertion	pfK13_K189T/N	Y184F	M74I	N75E	K76T	Patient CYP2D6
1							
2							
3	\	K	Y	M	N	K	
4	\	K	Y	M	N	K	
5							
6							
7							
8							
9							
10							
11							
12							
13							
14	\	K	Y	M	N	K	
15	\	K	Y	M	N	K	
16							
17							
18	\	K	Y	M	N	K	
19	\	K	Y	M	N	K	
20	\	K	Y	M	N	K	
21	\	K	Y	M	N	K	
22							
23							
24							
25							
26							
27							
28							
29							
30							
31							
32	\	K	F	M	N	K	
33	NN	K	F	M	N	K	
34	\	N	F	M	N	K	
35	\	N	F	M	N	K	
36	\	T	F	M	N	K	
37	\	T	F	I	E	T	
38							
39							
40							
41							
42	\	K	Y	M	N	K	
43	\	K	Y	M	N	K	
44							
45							
46							
47							
48							
49							

Sample	pfK13_NN insertion	pfK13_K189T/N	Y184F	M74I	N75E	K76T	Patient CYP2D6
50							
51							
52							
53							
54							
55							
56							
57							
58							
59							
60							
61							
62							
63							
64							
65							
66							
67							
68							
69							
70							
71							
72							
73							CYP2D6*2A，CYP2D6*36
74							
75							
76							
77							CYP2D6*4N，CYP2D6*2A
78							
79							
80							CYP2D6*4N，CYP2D6*15
81							
82							CYP2D6*2A，CYP2D6*4N
83							
84							
85							
86							
87							
88							
89							
90							
91							
92							
93							

Supplementary Table 2 List of PCR primers for amplicon deep sequencing of the seven markers.

Marker	Primer name	Sequence 5’-3’	Annealing (℃)	Amplicon length (bp)	GenBank	Reference
18S rRNA	*ssu_F*	GTGAAATTCTTAGATTTTCTG	58	304	LR131336	Lalremruata et al 2017
	ssu_R	CGTGTTGAGTCAAATTAAGC				
CYTB	*cytb_F*	GAGTGGATGGTGTTTTAGAT	54	344	KY923448	Lalremruata et al 2017
	cytb_R	GTGCTACCATGTAAATGTAA				
pfmsp1	*pfmsp1_F*	GAAGCTTTAGAAGATGCAGTATTGA	55	347	NC_004330	This study
	pfmsp1_R	TCAAAGAGTTCGGGATATTTGAG				
pfama1	*pfama1_F*	GAGTAGATTTAGGAGAAGATGC	50	390	KU863228	This study
	pfama1_R	GCTGTTTCTTTTACTTTCGTCT				
pvmsp1	*pvmsp1_F*	ACCCATACAAGCTGCTCGAC	60	309	AF435593	This study
	pvmsp1_R	GTGTTCACCTTGCTCACGAG				
pvama1	*pvama1_F*	TGGGTGCATTCAACTCGGAT	60	288	FJ785007	This study
	pvama1_R	GGTTCGCAGGGACATTTGAT				
pomsp1	*pomsp1_F*	GGAGATGTTAGATAAGGAGAAG	55	331	FJ824670	This study
	pomsp1_R	ATTTTGTCATTGTTGGGC				

Adapter for deep sequencing
Forward adapter: <u>TCGTCGGCAGCGTCAGATGTGTATAAGAGACAG</u>
Reverse adapter: <u>GTCTCGTGGGCTCGGAGATGTGTATAAGAGACAG</u>

Supplementary Table 3 Mean and median number of clustered reads of the major haplotypes within samples in the seven markers

Marker	Number of samples	Median	Min	Max	Mean	Std Err	95% Confidence Interval (CI)
CYTB	81	21416	149	24945	20069	633	18809-21330
SSU	85	12958	263	24797	11109	645	9828-12391
pfmsp1	49	8799	131	24988	11136	1208	8708-13564
pfama1	33	7642	108	24911	8656	1021	6577-10735
pvmsp1	14	9553	686	22520	9259	1818	5331-13186
pvama1	15	24895	222	24895	9250	1949	5069-13432
pomsp1	14	11267	2698	18057	10759	1495	7530-13988

Supplementary Table 4 The numbers of imported malaria cases and the origin of counties identified by amplicon deep sequencing.

Species	Ghana	Congo	Cameroon	Myanmar	Indonesia	Total	%
pf	34	10	6			50	53.8%
pv	6	7			1	14	15.1%
poc	6		3			9	9.7%
pow	3	1				4	4.3%
pm			1			1	1.1%
pf+poc	4	1	2	1		8	8.6%
pf+pv	2	1				3	3.2%
pf+pm				1		1	1.1%
pf+pow	1					1	1.1%
poc+pow	1					1	1.1%
pv+poc	1					1	1.1%
Total	58	20	12	2	1	93	100%

pf, *Plasmodium falciparum; pv, P. vivax; pm, P. malariae; poc, P. ovale curtisi;* and *pow, P. ovale wallikeri. Plasmodium* species were determined by amplicon deep sequencing of mitochondria *cytb* and *ssu* 18s ribosomal RNA genes.

Supplementary Table 5 Number of recurrent infections recrudescence, relapse, and parasite dormancy or presents in low parasitemia in the five malaria species.

Species	Parasite dormancy or presents in low parasitemia	Recrudescence	Relapse
P. falciparum	3	16	
P. malariae		1	
P. vivax		3 (1)	7 (2)
P. ovale curtisi		5 (4)	6 (3)
P. ovale wallikeri		1	3 (2)
Total	3	26 (5)	16 (7)

* Number in parentheses indicate the number of recurrent infections with inadequate anti-malarial treatment for relapse in previous malaria episode.

Supplementary Table 6 Additional cases in patients who had two tests within one week or had traveling abroad after first or second malaria attack.

Patient no.	Episode and interval (days)			Country of origin
	First	Second	Third	
Pat09	*pf* [#1]	*pf+pv* (3)		Ghana
Pat10	*pf* [#1]	*pf* (4)		Ghana
Pat13	*pf* [#1]+*pm*	*pf+poc* (6)		Myanmar
Pat02	*pf* [#1]	*pf* [#1] (22)	*pf* (366) [†]	Ghana
Pat43	*pf* [#1]	*pf* (167) [†]		Ghana, Cameroon
Pat28	*pf* [#1]	*pf* (401) [†]		Ghana
Pat06	*pf* [#1]	*poc* (461) [†]		Ghana
Pat38	*pv* [#3]	*pv* (503) [†]		Indonesia, Ghana
Pat05	*poc* [#3]	*pf* (696) [†]		Cameroon, Congo

pf, Plasmodium falciparum; pv, P. vivax; pm, P. malariae; poc, and *P. ovale curtisi;* mixed-species infections showed with "+", e.g. *pf+pv* represents mixed infections of *P. falciparum* and *P. vivax.* [†] represents patients who had traveling abroad after previous malaria attack.

Supplementary Table 7 Haplotype similarity of the 5 amplicons in comparing nucleotide sequences in NCBI GenBank databases.

Gene	*n*	Number of haplotypes	Predominant haplotypes			Other haplotypes		
			100%	98-100%	<98%	100%	98-100%	<98%
pfmsp1	49	60	11	15	1	7	21	5
pfama1	33	27	17	3		7		
pvmsp1	14	7	5	2				
pvama1	15	5	5					
pomsp1	14	5	1	1	3			

n, number of samples tested.

Supplementary Table 8 Mutation detections of the genes associated with antimalarial drug resistance of *P. falciparum* in the six patients with recurrent episodes. Amino acid mutation sites are indicated in bold italics. NN, N-terminal NN insertion between amino acids 136 and 137.

Patient	Episode	*pfmsp1* haplotype*	*pfK13*		*pfmdr1*	*pfcrt*		
			NN Ins.	K189T/N	Y184F	M74I	N75E	K76T
Pat02	1	M16M05	\	K	Y	M	N	K
	2	M16M08M05	\	K	Y	M	N	K
Pat07	1	M60M26M33M23M15M18	\	K	Y	M	N	K
	2	M60M05M03	\	K	Y	M	N	K
Pat09	1	M48	\	K	Y	M	N	K
	2	M48	\	K	Y	M	N	K
Pat10	1	M12M37M43M54M05	\	K	Y	M	N	K
	2	M12M37M43M54	\	K	Y	M	N	K
Pat16	1	M55	\	K	***F***	M	N	K
	2	M27	NN	K	***F***	M	N	K
Pat17	1	M01	\	***N***	***F***	M	N	K
	2	M01	\	***N***	***F***	M	N	K
Pat18	1	M42M58M49M39M46	\	***T***	***F***	M	N	K
	2	M49	\	***T***	***F***	***I***	***E***	***T***
Pat21	1	M01	\	K	Y	M	N	K
	2	M01	\	K	Y	M	N	K

* each "M" following a 2-digit number represents haplotype number; multiple haplotypes mean multiclonal infections and show in dominant haplotype first and least abundant haplotype in the end; mutation detection of minor clones in multiclonal infections is possible masked by drug sensitive parasites.
